# Supplementary material for: Bridging circuit modeling and signal analysis to understand the risk of crosstalk contamination in brain recordings
Source: Nat Commun. 2025 May 22;16:4744. doi: 10.1038/s41467-025-59391-0 (PMC12095702; doi:10.1038/s41467-025-59391-0)
Supplement: Supplementary file 1 — Supplementary Information [file 41467_2025_59391_MOESM1_ESM.pdf]

Bridging circuit modeling and signal analysis to understand the risk of crosstalk contamination in brain recordings

Maria F. Porto Cruz[1,5*], Elena Zucchini[5], Maria Vomero[1,2], Aldo Pastore[5,6], Ioana G. Vasilaş[1,2], Emanuela Delfino[5,6], Michele Di Lauro[6], Maria Asplund[1,2,4], Luciano Fadiga[5,6], Thomas Stieglitz[1,2,3*]

[1]Laboratory for Biomedical Microtechnology, Department of Microsystems Engineering (IMTEK) – University of Freiburg, 79110, Freiburg, Germany

[2]BrainLinks-BrainTools Center, University of Freiburg, 79110 Freiburg, Germany

[3]Bernstein Center Freiburg, University of Freiburg, Freiburg, Germany

[4]Freiburg Institute for Advanced Studies (FRIAS), University of Freiburg, Freiburg, Germany

[5]Department of Neuroscience and Rehabilitation, University of Ferrara, via Luigi Borsari 46, 44121 Ferrara, Italy

[6]Center for Translational Neurophysiology of Speech and Communication, Istituto Italiano di Tecnologia, via Fossato di Mortara 19, 44121 Ferrara, Italy

[*]Corresponding authors: mfportocruz@gmail.com, thomas.stieglitz@imtek.uni-freiburg.de

Supplementary information

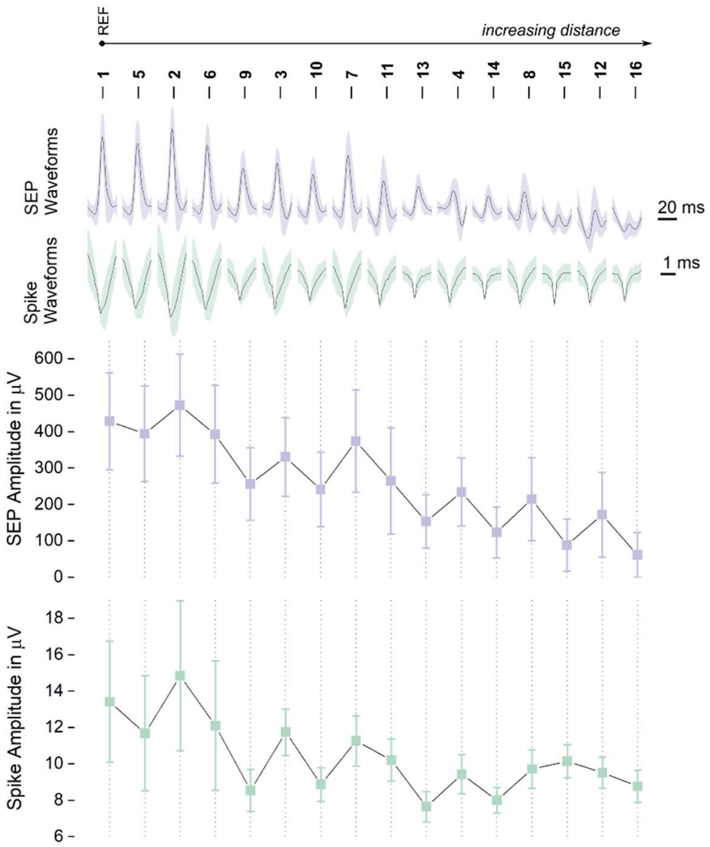

Figure S1: Signal amplitudes according to electrode position. Somatosensory evoked potential (SEP) waveform amplitudes and spike waveform amplitudes plotted for increasing interelectrode distance, taking electrode 1 as reference. Mean and standard deviation of SEP waveform amplitudes are calculated for n=60 with n as the total number of stimulus repetitions for one implanted device. Mean and standard deviation of spike waveform amplitudes are calculated for all detected spikes for each channel across the full recorded response. This is a detailed version of the data presented in figure 3.c.

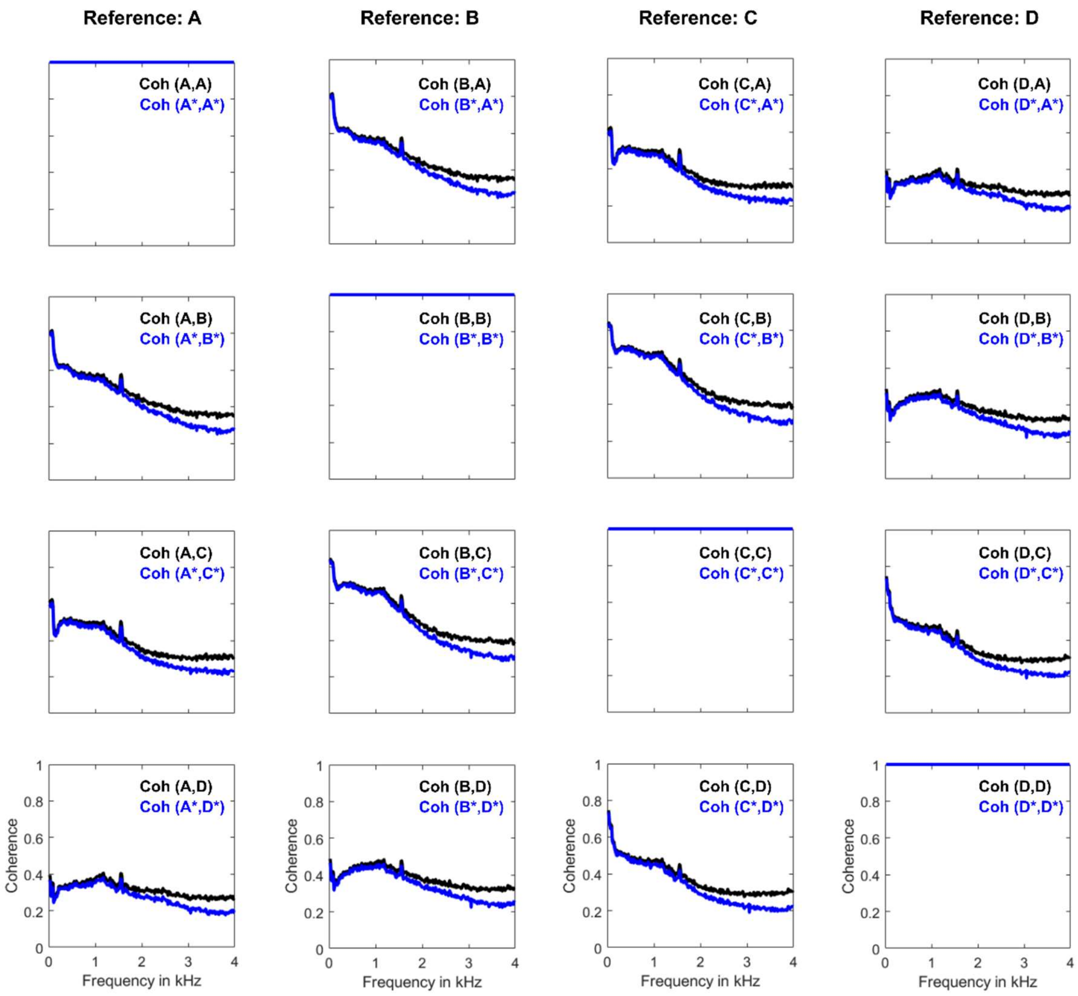

Figure S2: Coherence matrix before and after crosstalk back-correction. Coherence computed for a representative set of four channels A, B, C and D composing an electrode column of the electrode grid of one implanted device, taking each of the four electrodes as the reference. Signals after correction are symbolized by an asterisk (A*, B*, C* and D*).

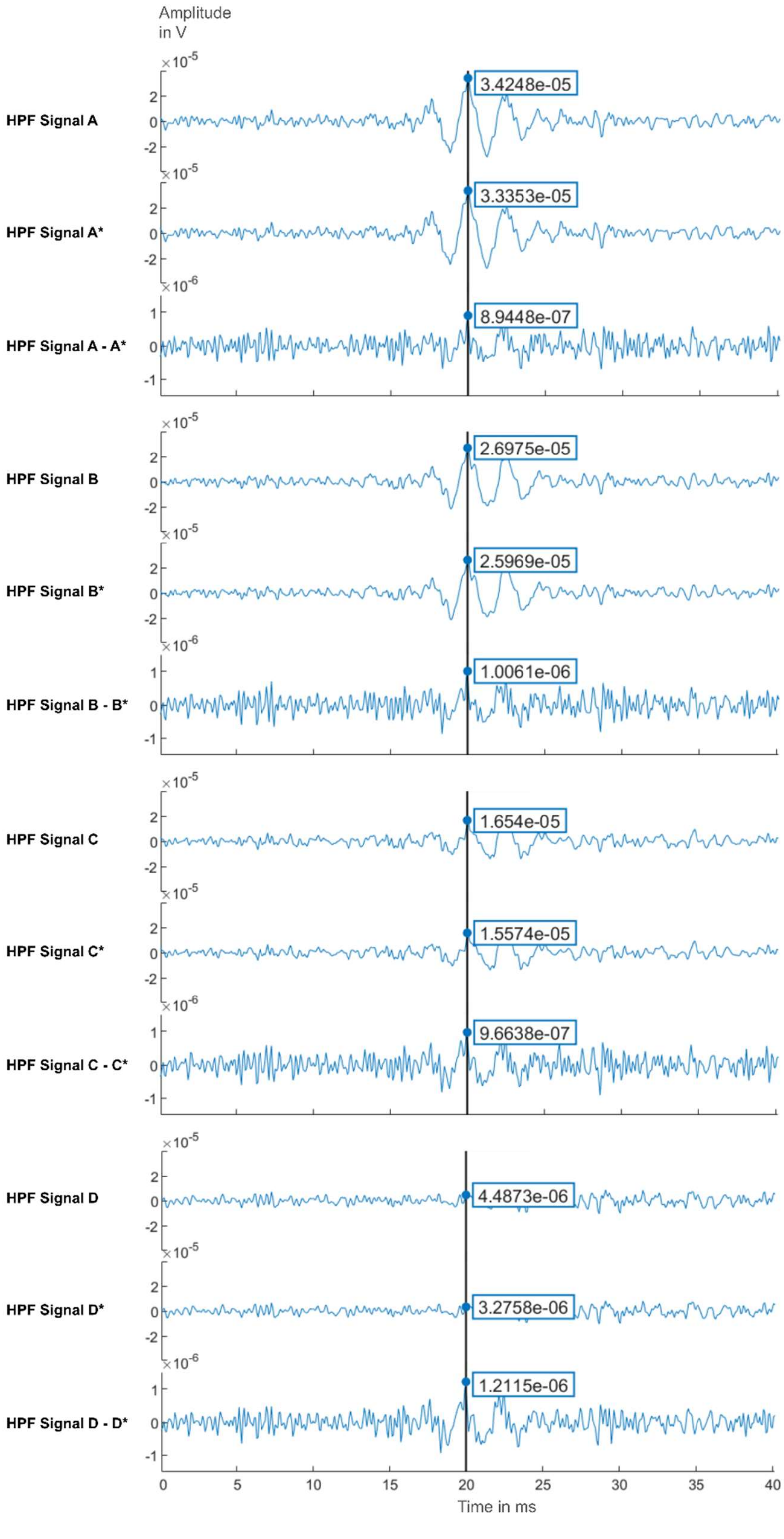

Figure S3: High-pass filtered signals before and after crosstalk back-correction. High-pass filtered (HPF) signals (fc = 300 Hz) computed for a representative set of four channels A, B, C and D composing an electrode column of the electrode grid of one implanted device. Signals after correction are symbolized by an asterisk (A*, B*, C* and D*).

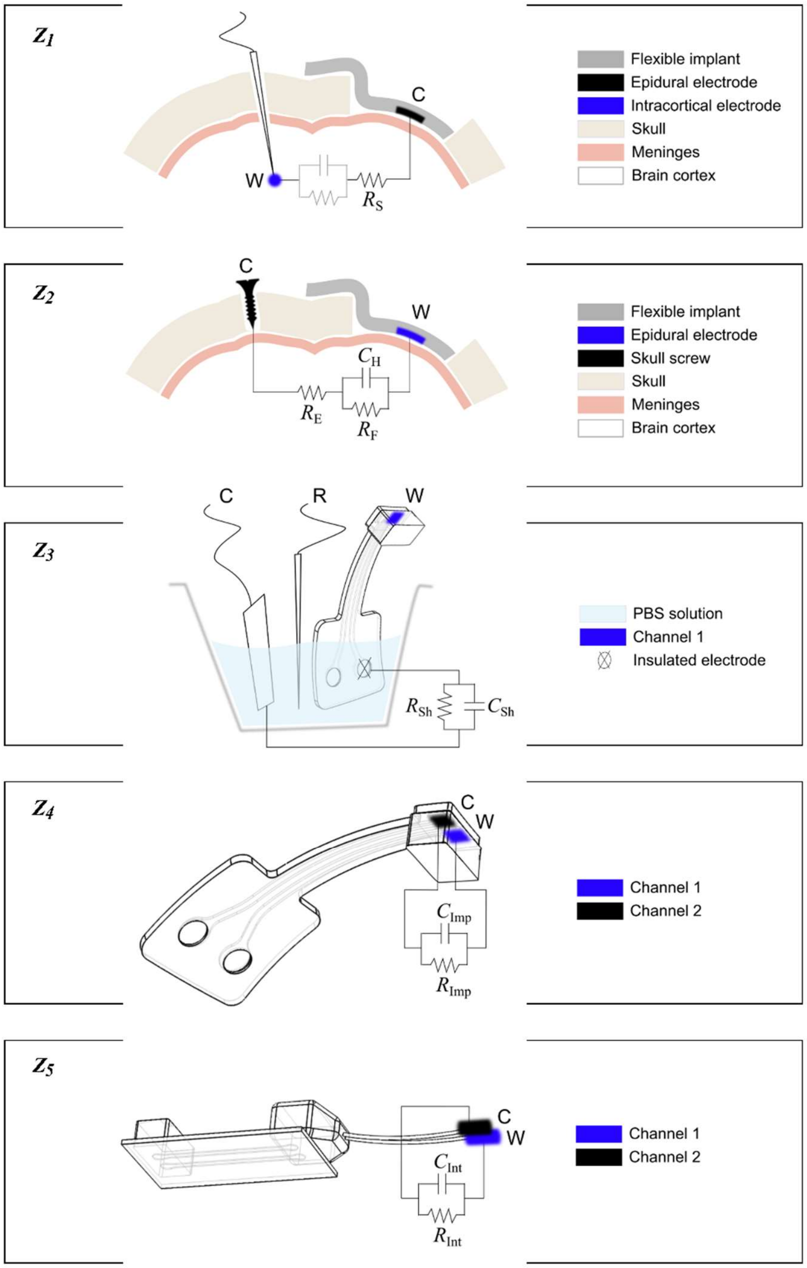

Figure S4: Impedance spectroscopy characterization. Set of experiments used to acquire the parameters of the transmission electrical model shown in figure 4. Resulting parameters specified in table 1. W, R and C stand for working, reference and counter electrode.

Table S1: Fitting analysis. New fittings for the experimental impedance spectroscopy data were performed in the software ZView (v4, Scribner, North Carolina, USA) to provide fitting errors and goodness of fit (chi-squared).

Block	Lumped-element	Symbol	Original Fitted Value (as per Table 1)	New Fitted Value	Deviation Factor	Fitting Error %	Chi-Squared	Method
1	Spread resistance	*R*S	45.3		N/A	N/A	N/A	Estimation of resistive component from magnitude curve above 0.1 MHz
2	Electrolyte resistance	*R*E	7.6	9.9	+0.303	12.548	0.0517	Fitting using ZView software
	Faradic resistance	*R*F	12	6.5	-0.458	14.502		
	Helmholtz capacitance	*C*H	4.4	1.9	-0.568	7.4186		
3	Shunt resistance to the electrolyte	*R*Sh	0.6	0.61	0.017	8.5435	0.0120	Fitting using ZView software
	Shunt capacitance to the electrolyte	*C*Sh	23	22.5	-0.022	4.2221		
4	Insulation resistance of the implant	*R*Imp	1.6		N/A	>100	N/A	Equivalent value given by impedance analyzer at 1 kHz
	Insulation capacitance of the implant	*C*Imp	4.8		N/A	41.86		
5	Insulation resistance of the interconnects	*R*Int	12.4	8.5	-0.315	47.978	0.0418	Fitting using ZView software
	Insulation capacitance of the interconnects	*C*Int	43.9	36.7	-0.164	7.7852		
6	Input resistance of the amplifier	*R*S	2.5		N/A	N/A	N/A	Specified by supplier
	Input capacitance of the amplifier	*R*E	8.8		N/A	N/A		
