# Supplementary material for: Bridging circuit modeling and signal analysis to understand the risk of crosstalk contamination in brain recordings
Source: Nat Commun. 2025 May 22;16:4744. doi: 10.1038/s41467-025-59391-0 (PMC12095702; doi:10.1038/s41467-025-59391-0)
Supplement: Supplementary file 2 — Reporting Summary [file 41467_2025_59391_MOESM2_ESM.pdf]

nature portfolio

Corresponding author(s): Maria Francisca Porto Cruz

Last updated by author(s): Mar 16, 2025

Reporting Summary

Nature Portfolio wishes to improve the reproducibility of the work that we publish. This form provides structure for consistency and transparency in reporting. For further information on Nature Portfolio policies, see our Editorial Policies and the Editorial Policy Checklist.

Statistics

For all statistical analyses, confirm that the following items are present in the figure legend, table legend, main text, or Methods section.

n/a	Confirmed	
☐	☒	The exact sample size (*n*) for each experimental group/condition, given as a discrete number and unit of measurement
☐	☒	A statement on whether measurements were taken from distinct samples or whether the same sample was measured repeatedly
☒	☐	The statistical test(s) used AND whether they are one- or two-sided<br>*Only common tests should be described solely by name; describe more complex techniques in the Methods section.*
☒	☐	A description of all covariates tested
☒	☐	A description of any assumptions or corrections, such as tests of normality and adjustment for multiple comparisons
☐	☒	A full description of the statistical parameters including central tendency (e.g. means) or other basic estimates (e.g. regression coefficient) AND variation (e.g. standard deviation) or associated estimates of uncertainty (e.g. confidence intervals)
☒	☐	For null hypothesis testing, the test statistic (e.g. *F*, *t*, *r*) with confidence intervals, effect sizes, degrees of freedom and *P* value noted<br>*Give P values as exact values whenever suitable.*
☒	☐	For Bayesian analysis, information on the choice of priors and Markov chain Monte Carlo settings
☒	☐	For hierarchical and complex designs, identification of the appropriate level for tests and full reporting of outcomes
☒	☐	Estimates of effect sizes (e.g. Cohen's *d*, Pearson's *r*), indicating how they were calculated

Our web collection on statistics for biologists contains articles on many of the points above.

Software and code

Policy information about availability of computer code

Data collection	Neural data were collected using a Tucker Davis Technologies multi-channel recording system 3 (Tucker Davis Technologies Inc., Florida, USA) including the ZIF-Clip® headstage with unity gain, the PZ2-256 battery-powered preamplifier and the RZ2 real-time processor. Two electrode Impedance data were recorded by potentiostat/galvanostat Reference 600 from Gamry Instruments Inc (Pennsylvania, USA) . Three-electrode impedance data were recorded using the potentiostat Autolab PGSTAT (Metrohm Autolab BV, Netherlands).
Data analysis	Neural Signal analysis was done with NeuroExplorer® software (v. 5.211, Nex Technologies, USA) and MATLAB workspace (v. R2019b, MathWorks, Massachusetts, USA. The transmission line was modeled in Simulink (v. R2019b, MathWorks, Massachusetts, USA).The transfer function was computed using the linear analysis tool and saved in the MATLAB workspace (v. R2019b, MathWorks, Massachusetts, USA). Impedance spectroscopy data were modeled in lumped parameter and fitting errors and goodess fit were calculated in the software ZView (v4, Scribner, North Carolina, USA). Fitting of two-electrode impedance data was done using the software Gamry Echem Analyst (v. 6,32, Gamry Instruments Inc, Pennsylvania, USA). Fitting of three-electrode recordings was done using the software Nova (Metrohm Autolab BV, Netherlands).

For manuscripts utilizing custom algorithms or software that are central to the research but not yet described in published literature, software must be made available to editors and reviewers. We strongly encourage code deposition in a community repository (e.g. GitHub). See the Nature Portfolio guidelines for submitting code & software for further information.

Data

Policy information about availability of data

All manuscripts must include a data availability statement. This statement should provide the following information, where applicable:

- Accession codes, unique identifiers, or web links for publicly available datasets
- A description of any restrictions on data availability
- For clinical datasets or third party data, please ensure that the statement adheres to our policy

All data supporting the findings of this study are available within the paper and its Supplementary Information.

Research involving human participants, their data, or biological material

Policy information about studies with human participants or human data. See also policy information about sex, gender (identity/presentation), and sexual orientation and race, ethnicity and racism.

Reporting on sex and gender	non applicable
Reporting on race, ethnicity, or other socially relevant groupings	non applicable
Population characteristics	non applicable
Recruitment	non applicable
Ethics oversight	non applicable

Note that full information on the approval of the study protocol must also be provided in the manuscript.

Field-specific reporting

Please select the one below that is the best fit for your research. If you are not sure, read the appropriate sections before making your selection.

☒ Life sciences ☐ Behavioural & social sciences ☐ Ecological, evolutionary & environmental sciences

For a reference copy of the document with all sections, see nature.com/documents/nr-reporting-summary-flat.pdf

Life sciences study design

All studies must disclose on these points even when the disclosure is negative.

Sample size	Neural recordings were collected from four anesthetized and head-fixed rats; Signals were time-locked to the start of whisker stimulation (n=60)
Data exclusions	none
Replication	Multiple animals (n=4) were used and a total of 60 trials was used to replicate data for hypothesis
Randomization	Randomization was not necessary since the technical system used in the study was subject of investigation and not the animals
Blinding	No blinding necessary since effects of the technical system were investigated and not the biological outcome which would need to take physiological or psychological effects into account.

Reporting for specific materials, systems and methods

We require information from authors about some types of materials, experimental systems and methods used in many studies. Here, indicate whether each material, system or method listed is relevant to your study. If you are not sure if a list item applies to your research, read the appropriate section before selecting a response.

Materials & experimental systems

n/a	Involved in the study
☒	☐ Antibodies
☒	☐ Eukaryotic cell lines
☒	☐ Palaeontology and archaeology
☐	☒ Animals and other organisms
☒	☐ Clinical data
☒	☐ Dual use research of concern
☒	☐ Plants

Methods

n/a	Involved in the study
☒	☐ ChIP-seq
☒	☐ Flow cytometry
☒	☐ MRI-based neuroimaging

Animals and other research organisms

Policy information about studies involving animals; ARRIVE guidelines recommended for reporting animal research, and Sex and Gender in Research

Laboratory animals	Long Evans rats with 400-500 g
Wild animals	no
Reporting on sex	lack of sex-based analysis since physical properties of the technical system are subject of research and not potential variances in the biological signal
Field-collected samples	not-applicable
Ethics oversight	Ethics Committee for animal research of the University of Ferrara and by the Italian Ministry of Health (authorization n. 989/2020-PR)

Note that full information on the approval of the study protocol must also be provided in the manuscript.

Plants

Seed stocks	not used
Novel plant genotypes	not used
Authentication	not applicable
